# Comments on the paper “Data-driven gating (DDG)-based motion match for improved CTAC registration. *EJNMMI Physics.* 2024;11(1):42.”

**DOI:** 10.1186/s40658-024-00690-8

**Published:** 2024-10-21

**Authors:** Tinsu Pan

**Affiliations:** Department of Imaging Physics, University of Texas, M.D. Anderson Cancer Center, Houston, TX USA

**Keywords:** Misregistration of PET and CT, Data-driven gated PET, 4D CT, Data-driven gated CT

## Abstract

Misregistration between CT and PET in PET/CT is mainly caused by respiratory motion or irregular respiration during the CT scan in PET/CT. Other than repeat CT, repeat PET/CT, or data-driven gated (DDG) CT, there is no practical approach to mitigate the misregistration artifacts and subsequent CT attenuation correction (CTAC) of the PET data. DDG PET derives a respiratory motion model based on the multiple phases of PET images without hardware gating and it allows for a potential correction of the misregistration artifacts based on the respiratory motion model. The purpose of this commentary was to compare the recent two publications on matching the random phase of helical CT with one of the PET phases derived from the motion model of DDG PET and warping the misregistered helical CT for CTAC of and registration with PET or DDG PET. The two publications were similar in methodology. However, the data sets used for the comparison were different and could potentially impact their conclusions.

## Background

Misregistration between CT and PET in PET/CT is mainly caused by respiratory motion or irregular respiration during the CT scan in PET/CT. Other than repeat CT, repeat PET/CT, or data-driven gated (DDG) CT, there is no practical approach to mitigate the misregistration artifacts and subsequent CT attenuation correction of the PET data. CT in PET/CT is normally acquired at minimum from the orbit to the mid-thigh. Repeat CT imposes a significant CT dose penalty. Repeat PET/CT of one to two bed-positions of the misregistration area reduces CT radiation dose but significantly increases the acquisition time because of repeat PET. DDG CT can register with DDG PET, but it requires a cine CT scan of about 1.3 mSv [1]. DDG CT is more reliable than repeat CT or repeat PET/CT because it can derive average CT and end-expiration phase CT to match with PET and DDG PET, respectively [1]. DDG PET allows for a potential correction of the misregistration artifacts based on the respiratory motion model derived from the multiple phases of PET without hardware gating. The purpose of this commentary was to compare the recent two publications on matching the random phase of helical CT with one of the PET phases derived from the motion model of DDG PET and warping the misregistered helical CT for CTAC of and registration with PET or DDG PET. Both approaches do not require additional CT scans.

## Main text

We wish to comment on the recent publication on the correction of misregistration between PET and helical CT in PET/CT [2], which was similar in concept to our publication [3]. The root cause of misregistration stems from a mismatch of the temporal resolutions of sub-second CT images and average PET which is acquired over many breathing cycles of about 3 to 5 s. In other words, it is caused by the difference between fast CT and slow PET scans of a free-breathing patient. It is a problem mainly with CT rather than PET as CT takes snapshots of the anatomy in respiration and in translation by the patient table whereas PET is averaged over many breathing cycles. Both papers suggested the potential of matching the random phase of helical CT with one of the PET phases derived from the motion model of DDG PET and warping the misregistered helical CT for CTAC of and registration with PET or DDG PET. However, there were differences in the selection of patient data and gold standard CT used in the comparison.

The inclusion criterion of patient data in Cook’s paper was based on the R-value calculated in the Fourier domain using respiratory waveforms where the R-value is the ratio of the peak value within the respiratory frequencies (0.1–0.4 Hz) from each principal component to the mean value beyond this frequency range [2]. This inclusion was solely based on the respiratory motion detected in the DDG PET. Misregistration between CT and PET can still occur when DDG PET detects little motion or R-value does not exceed a threshold. This selection could miss some misregistered PET/CT data and lead to less misregistration in the selected data. The inclusion criterion in Sun’s paper was based on an identified misregistration between helical CT and PET by the technologist. This selection is relevant to the clinical PET/CT acquisition when PET/CT data is reviewed for data integrity before patient release. The difference in the selection criterion was evident in the extent of the motion modeled for warping or deformable image registration (DIR): 200% and 400% of the amplitude range from end-expiration (EE) at 0% to end-inspiration (EI) at 100% detected in DDG PET in Cook’s and Sun’s, respectively. There was less misregistration between CT and PET in the patient population of Cook’s than in Sun’s, and this might make the results in Cook’s better than the ones in Sun’s as there was less DIR required for the data of less misregistration. Nevertheless, the use of DIR was found to cause a bubbling liver artifact [2], and a severe distortion of the anatomy in CT, particularly for the patient studies whose misregistered helical CT matched a PET phase of greater than 250% of the amplitude range of EE to EI [3].

There was another difference between 4D CT and DDG CT as the gold standard in Cook’s and Sun’s, respectively. Both 4D CT and DDG CT were derived from the cine CT acquisition over a respiratory cycle. 4D CT, but not DDG CT, requires an additional gating device on the patient. The radiation dose of 4D CT to helical CT was 5 times in Cook’s and the radiation dose of DDG CT to helical CT was 1 time in Sun’s. A lower dose DDG CT was chosen primarily for CTAC and localization. Cine CT was acquired for 4D CT before the acquisition of PET/CT of 1-bed position regardless of the extent of misregistration. In contrast, cine CT for DDG CT was only acquired when there was misregistration between helical CT and PET and the scan coverage of cine CT was adjusted to cover the extent of misregistration. DDG CT was better than 4D CT in the appropriate selection of the EE and EI phases of CT [1]. This was because the EE and EI phases of DDG CT can be determined “internally” from the CT number measurement of the lung regions and the variation of the body outlines in the cine CT images. In 4D CT, the EE phases were chosen “externally” either from the mid-point or near the mid-point between the two consecutive EI phases determined by external device gating, and the EI phases sometimes cannot be determined accurately in a prospective manner and require the operator’s manual selection [4].

Both used the same DIR and assumed a linear respiration model to DIR the misregistered helical CT to PET or DDG PET. The misregistered helical CT was matched to one PET phase with mutual information in Cook’s and with line profiles in Sun’s. This model seemed to work well for CTAC but less so for localization of a tumor in particular when there was a severe misregistration of greater than 250% of the amplitude range of EE to EI or the tumor was near the boundaries of two organs such as the lung/ribs, the lung/liver, and the liver/gallbladder etc [3]. Distortion of anatomy coupled with blurry appearance or lower resolution DIR CT could also potentially lower the confidence of a radiologist or nuclear medicine physician in reading the PET/DIR CT data. For practical purposes, DDG CT of the radiation dose of about 1.3 mSv is used in our clinic to correct for misregistration of CT with PET or DDG PET once a misregistration is identified. The radiation dose of DDG CT was about 35% of the dose of the repeat CT in repeat PET/CT [5]. This has served us well in misregistration correction when most patients are covered with a warm blanket during the PET/CT scan, which makes external gated 4D CT difficult or impossible to apply in the clinic [1].

## Conclusions

Both approaches seemed to work well for CTAC but not for tumor localization particularly for a severe misregistration or a tumor near the boundaries of two organs. More research in DIR or artificial intelligence is warranted to realize misregistration-free PET/CT for clinical practice without repeat CT, repeat PET/CT, or DDG CT.

## Abbreviations

DDG: Data-driven gated/gating
DIR: Deformable image registration
EE: End-expiration
EI: End-inspiration
4D: Four dimension
